# Medical Opinion and Movement

**Published:** 1910-08-20

**Authors:** 


### Medical Opinion and Movement.

### Ossified Costal Cartilages and the X-rays.

In the *Münchener Medizinische Wochenschrift* Dr. Wiesner reports some observations on workers in *x*-ray laboratories from which it would appear that the *x*-rays tend to produce a premature ossification of the costal cartilages. In four individuals aged 28, 32, 34, and 45 employed for several years (seven to fourteen) in *x*-ray laboratories he has found this premature ossification of all the costal cartilages. On radiological examination the first cartilage appeared to be completely ossified and gave as deep a shadow as that of the rib itself. The ossification of the other cartilages was more or less marked and had followed an irregular process of development, sometimes chiefly involving the lower border and sometimes the upper, and sometimes both like the prongs of a fork. On examining radiographs of thoraces from individuals of about the same age, whc had not been continually exposed to the *x*-rays, Dr. Wiesner has failed to find the same characteristic ossification. Some of them showed more or less ossification of the first cartilage, but the remaining cartilages were quite free. The author is therefore of opinion that this premature ossification is directly attributable to the continued action of *x*-rays.

### Gastric Crises of Tabes.

Reference has already been made in these columns to the treatment of lightning pains and other morbid affections of tabes dorsalis of the lower part of the body by division of the posterior nerve roots. Similarly for the gastric crises of extreme severity Foerster has proposed the division of the seventh to the ninth dorsal posterior roots on the supposition that the crises are due to irritation of the sympathetic nerve fibres in the stomach. Eppinger and Hess ascribe their cause to an irritation of the nerve endings by a tabetic toxin. In either case, as Goetzl explains, section of the sympathetic fibres would interrupt the reflex arc from the sensory sympathetic to the motor vagus fibres, and so prevent the occurrence of the crises. Whatever be the explanation, the fact remains that in the few cases reported in which the operation has been carried out, marked relief appears to have followed. Küttner reports a case in which the operation was performed as a *dernier ressort*, and the patient was completely relieved of his suffering. A similar successful case is reported by Goetzl. Other cases are reported by Bruns and Sauerbruch. In conditions in which there is also intestinal colic they advise inclusion of the tenth dorsal in the section. Although one would *à priori* hesitate to submit tabetic patients to such an operation except in view of extremely distressing symptoms which it is sought to relieve, yet according to reports the operation has been well borne and even followed by a gain in weight in advanced cases of the disease.

### Alcohol Disinfection of Hands.

According to the observations of Professor Schumburg a surgeon on the general staff of the German Army reported in the *Deutsche Medizinische Wochenschrift* washing the hands with strong alcohol is a most effective means of removing all infection and rendering any bacteria innocuous. This author states that 200 c.cm. of alcohol applied with a pledge of cottonwool are sufficient to disinfect the hands to the extent of 99 per cent. or more of all bacteria present. Ordinary methylated spirit is quite effective. From experiments in the medical department of the Prussian Ministry of War it appears that washing with soap and water combined with even prolonged scrubbing with a brush does not remove the microbes, the soap softening the skin and making the bacteria more adherent. Alcohol, on the other hand, by hardening the skin, causes the bacteria to become rapidly detached. To secure proper disinfection with alcohol the preliminary use of soap and water must be dispensed with, the reasons given being that the residual moisture, even after drying, dilutes the alcohol, and further that the softening of the skin by water causes it to contract too strongly when the alcohol is applied, and by rendering it rough and scaly encourages the transference of bacteria from the surgeon's hands to the wound. Inasmuch, however, as the wearing of gloves for operations has been now so generally adopted, disinfection of the hands has not the same importance that it once possessed for the success of aseptic surgery.

### Gastric Radiology.

Most radiological observations on the stomach have been carried out with the subject in the upright position. Drs. Markovic and Perussia have conducted their examinations with the patient lying down, and they find a considerable difference in the motor activity of the stomach according to whether the patient lies on the right side or the left. An account of these experiments appears in the *Medizinische Klinik*. In a normal individual, if repeated observations are made after a bismuth meal taken fasting, so as to determine the exact time when the stomach contains no further trace of bismuth, they find that the time for evacuation varies from two and a half to four and a half hours for the right side, and five to eight hours for the left. In morbid conditions of the stomach these relative differences show considerable variations, so that such observations appear to possess some diagnostic value. Thus a greater difference in time of evacuation between the two positions, due to a greater rapidity of evacuation with the patient lying on the right side is in favour of pyloric insufficiency. On the other hand, a smaller difference between the two sides in consequence of greater rapidity of evacuation for the left side points to hypermobility. In conditions of atony with motor insufficiency the diminished muscular force is much more apparent and the evacuation much slower with the patient on the left side, and the difference between the two positions is therefore much increased. In pyloric stenosis in the early stages on the other hand, the difference between the two sides is less, as the muscular force is rather increased and the obstruction at the pylorus remains the same for either position. From these observations, too, it is advisable for those suffering from gastric atony to lie on the right side.

### Congenital Blue Spot in Mongols.

A peculiar oval bluish spot situated over or a little above the sacrum in children of the Mongolian race, or whose blood is partially derived from Mongolian sources, is described by Dr. E. Apert in *La Presse Médicale*. It is rarely seen in European and never in Negro children. It is caused by a deposit of black pigment in the deeper layers of the cells of the derma. It does not disappear on pressure. The author calls attention to it so that the practitioner may be in a position to reassure the parents of such children that it is not a true abnormality, that it has no pathological significance, and that before the child has reached the age of puberty it will have disappeared spontaneously.

### The Genital Crisis of the New-born.

This condition consists according to Lequeux and Marioton, who write in the *Bulletin de la Société d'Obstétrique de Paris*, in a swelling of, and a secretion of a milky fluid by, the mammary glands in both sexes. In the male in addition there may be swelling of the testes and of the prostate gland, together with a secretion of fluid in the tunica vaginalis. In the female the uterus may be congested and menstruation occur. Acne, seborrhœa, and an increase in lanugo may be noticed in both sexes. The phenomenon was met with in 532 out of 1,575 cases in the Tarnier clinique, and seemed commoner in well-developed than in premature and weakly infants. Hydrocele occurred in 67, menstruation in five cases. The crisis occurs most commonly about the tenth and is rare before the seventh day following delivery. Both sexes would seem to exhibit the phenomenon in equal proportions. It is purely physiological and needs no treatment as it is not an inflammatory process. The mammary secretion is composed of fat globules and epithelial cells, and no micro-organisms are found in it, unless infected from without by manipulation. The crisis is possibly caused by some internal secretion of the mother's placenta.

### The Coagulation Time of Blood.

The *Berliner Klinische Wochenschrift* publishes an article by Werner Schultz on a new method of estimating the coagulation time of the blood. The writer claims for it greater simplicity and greater rapidity of action than is the case with the numerous methods previously devised for the purpose. The apparatus consists of four inches of capillary tubing blown out into a dozen or more small bulbs. Blood is drawn into the apparatus until this is completely filled. The bulbs are then broken off at fixed intervals of time one after the other, and shaken in test-tubes containing normal salt solution. It will then be seen that uncoagulated blood will be at once shaken out of the bulb, whereas coagulated blood will remain inside as a clot. The author claims that this procedure has the advantage over Wright's method in that only a few tubes are handled, and no elaborate system of time-keeping is necessary. It has, however, the disadvantage that a new tube is required for each experiment.

### Inoculation of Guinea-pigs.

Hoffmann in the *Deutsche Med. Woch.* reports the results of some experiments he has made on guinea-pigs in attempting to inoculate them with the spirochæte of syphilis. Rabbits have recently been successfully inoculated, and in his experiments he made use of tissue obtained from a syphilitic tumour of the testis of a rabbit. The testes of three guinea-pigs were inoculated with this tissue, and fourteen days later an ulcer appeared at the site of inoculation with a dirty base, thickened and indurated edges. Five days later the ulcer showed signs of healing, but the induration persisted. Large numbers of the Treponema pallidum were observed in the secretions from the ulcer.

### Inflammatory Intestinal Tumours.

According to H. Braun in the *Deutsche Zeitsch. f. Chirurgie*, the fact that inflammatory tumours of the intestine have not often been described is due either to their being overlooked or to their having been excised as malignant without subsequent microscopic examination. In his paper the author does not deal with tumours resulting from syphilis, tubercle, or actinomycosis, but only with those of the intestinal wall which are sessile, immovable, inflammatory, and circumscribed. Their ætiology is often obscure, but in most cases they result from an inflammatory process of the mucosa, which extends outwards into the fatty connective tissue of the mesocolon. Occasionally they result from extension from neighbouring parts. Frequently slight mucosal changes are not characterised by any morbid symptoms, so that the development of the tumour passes unnoticed, and eventually the mass is mistaken for carcinoma. This is more common in the aged, and when developed the tumour causes symptoms very similar to those of a malignant growth, and cachexia even is observed. Moreover, some of these tumours recur after removal. Diagnosis will then depend entirely on the microscope. The course of these tumours varies very much. Frequently they grow to a considerable size, cause obstruction, and need to be removed. At times, after reaching a certain size, they gradually become smaller and finally disappear. This spontaneous cure explains how a fæcal fistula resulting from a supposed carcinoma closes up by itself after a time, and, moreover, it explains how, as the result of some secret remedy or of faith-healing, a case diagnosed as inoperable carcinoma will at times recover spontaneously. Diagnosis can only be established by laparotomy or by a definite history of inflammatory trouble, but a febrile condition favours the diagnosis of inflammatory tumour. The surgeon when operating should have this form of growth in mind, and, if necessary, incise it and examine the cut edge. Treatment is not well defined. If diagnosis is fairly certain it is well to delay operation to see if the mass will disappear under treatment. If, however, it is doubtful, an exploratory operation should be performed to exclude a malignant growth. When there are no symptoms of obstruction it may be sufficient to remove an inflammatory tumour down to the level of the intestinal wall, but recurrence is then apt to take place. Entero-anastomosis is always preferable if the conditions allow of its being done, especially if there be obstruction or increased or painful peristalsis.

### A Nicotine Skin Eruption.

A case of extensive erythema following poisoning by nicotine is reported by Professor Näcke in the *Münch. Med. Woch.* The patient, by trade a joiner, was admitted into an asylum in a maniacal condition which had lasted some three weeks. He had experienced a similar, but milder, attack two years previously from which he practically recovered completely. Two weeks after admission he drank a bowl of coffee to which 7 to 10 grams of chewing tobacco had been added by a patient whose coffee he was in the habit of stealing. Next morning he vomited repeatedly. A general, slightly raised, scarlatiniform eruption appeared, especially on the back and buttocks, accompanied by severe itching, the patient being isolated for suspected scarlet fever, although his temperature was only 99°. Next day the eruption had spread further to the face, but was practically absent from the legs, forearms, hands, feet, and flexor surfaces of the joints. The face was puffy, the conjunctivæ hyperæmic, pupils contracted and reacted sluggishly to light and accommodation. There was nausea and severe headache. The pulse, somewhat irregular, ranged between 120 and 130. The temperature was normal, as was the urine. On the third day the eruption began to fade. Dark bluish-red rings enclosed normal skin on the back, and the lower lids resembled bluish œdematous bags. The patient had moderate headache, but complete anorexia. His pulse was still 120 and irregular. During the day he became more sensible. On the following day the rash had further faded, appetite returned, and the puffy eyelids improved. The pulse fell to 112 and the pupillary reactions returned to normal. Next day the pulse was 76 and the rash had disappeared, there being no desquamation. The maniacal condition gradually returned as the effect of the poisoning wore off. The author thinks that such an extensive erythema is probably unique after nicotine poisoning, though patches of erythema have been previously reported by Lewin as having occurred on the face and neck with pruritus on the neck, sternum, and between the scapulæ.

### Solid Carbon Dioxide for Rodent Ulcer.

Dr. Reginald Morton gives a preliminary account of the progress he has made with the employment of solid carbon dioxide for three selected cases of rodent ulcer. He has confined himself so far to cases of simple, uncomplicated ulcer, not too near the eye, nose, or mouth. He first scrapes away the granulation tissue until a firm but raw surface is left, then takes a solid carbon-dioxide crayon trimmed to an end very slightly larger than the ulcer, and applies it with firm pressure for about forty seconds. A lively reaction with exudation follows, and a boracic acid ointment is applied. In a few days a healthy healing sore is in evidence, which rapidly skins over into a soft elastic scar hardly noticeable except to a practised eye. The first case has now been healed for three months and shows no tendency to relapse. Reports of the permanence of this cure, and of the possibility of extending the method to more complicated cases, will no doubt be forthcoming in due time.

### Delayed Menopause.

The dangers of delayed menopause, and the therapeutic measures indicated in treating this condition, are discussed in the *New York Medical Journal* by Dr. A. E. Callant. He presents a table showing the approximate age at which menopause should be established, from which he argues that when the menses commence at the tenth they should end at the fiftieth to the fifty-second year, and when they start at the twentieth, the thirtieth to the thirty-second year should see the commencement of the menopause. In general, the menopause should come on abruptly, at an age corresponding to that at which the menses started in a woman with healthy reproductive organs, that is, early if the menses came on late and late if they came on early. Some diseased condition is present, such as version, flexion, fixation, neoplasm, tubal disease, syphilis, etc., when menstruation continues beyond the estimated time. The danger of a delayed menopause lies in the presence or promotion of fibroid or malignant tumours. Contributory dangers lie in the patient failing to seek or refusing advice, delay on the part of the physician to examine, or failure on his part to diagnose the case, and the putting off of the operation in the hope that time will relieve the symptoms. The danger signals in delayed menopause are atypical menstruation, hæmorrhage, evil-smelling discharge, and pain. When a woman has reached the age at which the menopause should be expected, there can be no question as to the justifiability of removing a useless, probably dangerous, tumour-breeding organ, and removal is the only sure method of putting an end to all dangers. The large number of incurable cases of carcinoma uteri, degenerated fibroids, ovarian disease, etc., occurring even in early life makes it imperative for the physician to instruct the mothers of families on the dangers of delay when their daughters suffer from dysmenorrhœa, menorrhagia, metrorrhagia, leucorrhœa, abdominal tumours, etc., and on the dangers to themselves when such symptoms occur at an age when the menopause should have been established.

